# The role of LDH serum levels in predicting global outcome in HCC patients treated with sorafenib: implications for clinical management

**DOI:** 10.1186/1471-2407-14-110

**Published:** 2014-02-20

**Authors:** Luca Faloppi, Mario Scartozzi, Maristella Bianconi, Gianluca Svegliati Baroni, Pierluigi Toniutto, Riccardo Giampieri, Michela Del Prete, Samuele De Minicis, Davide Bitetto, Cristian Loretelli, Marco D’Anzeo, Antonio Benedetti, Stefano Cascinu

**Affiliations:** 1 Department of Medical Oncology, Translational Oncology Unit, AOU Ospedali Riuniti, Università Politecnica delle Marche, Via Conca 71, 60126 Ancona, Italy; 2 Clinica di Gastroenterologia, AOU Ospedali Riuniti, Università Politecnica delle Marche, Ancona, Italy; 3 Internal Medicine, Department of Medical Sciences Clinical and Experimental, University of Udine, Udine, Italy; 4 Cancer Genetics Program, Departments of Medicine and Pathology, Beth Israel Deaconess Medical Center, Boston, MA, USA

**Keywords:** Hepatocellular carcinoma, Lactate dehydrogenase, Sorafenib, Angiogenesis

## Abstract

**Background:**

In many tumour types serumlactate dehydrogenase (LDH) levels proved to represent an indirect marker of tumour hypoxia, neo-angiogenesis and worse prognosis. As we previously reported LDH is an important predictive factor in hepatocellular carcinoma (HCC) patients undergoing transarterial chemoembolization (TACE). Sorafenib represents the therapeutic stronghold in advanced HCC patients. As a tyrosine kinase inhibitor (TKI) mainly directed against the angiogenetic pathway, the correlation of sorafenib administration with markers of hypoxia could be an important tool in patients management. Aim of our analysis was to evaluate the role of LDH pre-treatment levels and its variation during treatment in HCC patients receiving sorafenib.

**Methods:**

78 patients were available for our analysis. For all patients LDH values were collected within one month before the start of treatment and after the end of therapy. For study purposes we divided our patients into two groups, according to LDH pre-treatment levels, cut-off levels was determined with ROC curve analysis. Patients were, also, classified according to the variation in LDH serum levels pre- and post-treatment (increased vs decreased).

**Results:**

Patients proved homogeneous for all clinical characteristics analyzed. In patients with LDH values under the cut-off median progression free survival (PFS) was 6.7 months, whereas it was 1.9 months in patients above the cut-off (p = 0.0002). Accordingly median overall survival (OS) was 13.2 months and 4.9 months (p = 0.0006). In patients with decreased LDH values after treatment median PFS was 6.8 months, and median OS was 21.0 months, whereas PFS was 2.9 months and OS 8.6 months in patients with increased LDH levels (PFS: p = 0.0087; OS: p = 0.0035).

**Conclusions:**

In our experience, LDH seemed able to predict clinical outcome in terms of PFS and OS for HCC patients treated with sorafenib. Given the correlation between LDH levels and tumour angiogenesis we can speculate that patients with high LDH pretreatment levels may be optimal candidates for other emerging therapeutic agents or strategies targeting different molecular pathways.

## Background

Hepatocellular carcinoma (HCC) represents the commonest primary cancer of the liver. Incidence is increasing and HCC has risen to become the 5th commonest malignancy worldwide and the third leading cause of cancer related death, exceeded only by cancers of the lung and stomach [1]. Surgery is the only potentially curative treatment for HCC. In carefully selected patients, resection and transplantation in fact, allow a 5 years survival ranging from 60 to 70%, and should be considered as a first treatment option in this setting [1].

Unfortunately most patients in Western countries present with an advanced HCC at diagnosis with the consequent impossibility to use curative treatments. In these patients prognosis is poor with a median survival of less than 1 year [1].

In the last few years the introduction of sorafenib, an oral multi-tyrosine kinase inhibitor (TKI) for the treatment of advanced HCC patients changed the clinical landscape for these tumours and now represents the standard of care [2-4].

However a large proportion of patients still does not seem to benefit from such a treatment approach and are therefore exposed to unnecessary toxicity [2-4].

Clinical or molecular criteria allowing a more accurate selection of resistant/responder tumours are in fact largely lacking, although they would be obviously crucial for an optimal management of these patients in the clinical practice [5].

Hypoxia represents a clinical biological mechanism for treatment resistance in cancer cells via the formation of new blood vessels. Furthermore, a growing body of evidence indicates that hypoxia might actually promote cancer development. Lactic dehydrogenase (LDH), which is a glycolytic enzyme, composed of four polypeptide chains, each one encoded by separate gene (M and H), exists in various types of human tissue and neoplasms. LDH is a key enzyme in the conversion of pyruvate to lactate under anaerobic conditions [6]. Five isoforms of LDH have been identified as a result of the five different combinations of polypeptide subunits [7]. In preclinical models up-regulation of LDH has been suggested to ensure both an efficient anaerobic/glycolytic metabolism and a reduced dependence on oxygen under hypoxic conditions in tumor cells. The biological link between hypoxia, LDH levels and the tumor-driven angiogenesis pathway through the abnormal activation of the hypoxia inducible factor 1 (HIF-1) is well established. The biological activity of HIF-1 is determined by the expression and activity of the HIF-1α subunit [8]. HIF-1α is an essential factor that up regulates a series of genes involved in glycolytic energy metabolism, angiogenesis, erythropoiesis and cell survival [9]. Hypoxia in the tumor microenvironment is sufficient to activate HIF-dependent expression of several downregulated genes [10]. These include genes encoding for vascular endothelial growth factor, erythropoietin and many enzymes involved in glucose, iron, and nucleotide metabolism [11].

Although links among these factors are well known, their translation into clinical practice is still poorly investigated. The aim of our analysis is to assess the role of LDH serum concentration in a population of advanced HCC patients, treated with sorafenib.

## Methods

### Patients selection

This is a retrospective multicentre analysis. Two centres in Italy (Translational Oncology Unit and Gastroenterology Department, AOU “Ospedali Riuniti” – Università Politecnica delle Marche, Ancona; Internal Medicine, Department of Medical Sciences Experimental and Clinical - Università di Udine, Udine) were involved in the study.

From 2008 to 2012, consecutive patients with advanced HCC or intermediate stage HCC refractory to or unsuitable for locoregional therapies, either histologically proven or diagnosed according to the AASLD guidelines (American Association for the Study of Liver Diseases 2005) and receiving sorafenib were eligible for our analysis. All patients received sorafenib with standard schedule (400 mg bid continuously) dose reduction was applied as clinically indicated. Follow-up consisted of physical examination, a complete blood count, alpha-fetoprotein (α-FP) assay, computed tomography or magnetic resonance imaging (CT/MRI) scanning as clinically indicated. Tumour response was evaluated every 8 weeks by clinicians’ assessment imaging and according to the modified Response Evaluation Criteria in Solid Tumours (mRECIST) [12]. Radiological images were reviewed in double-blind by two radiologists.

Patients were classified according to ECOG PS (Eastern Cooperative Oncology Group performance status) and were staged using Child-Pugh and BCLC (Barcelona Clinic Liver Cancer) classifications.

In order to investigate whether LDH might be used as an early predictor of sorafenib failure we recorded LDH serum levels pre- (within 1 month prior the start of sorafenib treatment) and post-treatment (within one month after the end of sorafenib treatment). LDH serum levels were determined according to IFCC (International Federation of Clinical Chemistry and Laboratory Medicine) method. The assay has been conducted in Institution Laboratories certified for Quality control according to the present rules in Europe (ISO 9001:2008). The study received clearance by the local Ethical Committee.

### Statistical analysis

Statistical analysis was performed with MedCalc software version 10.4.8 for Windows. Patients were divided into two groups, according to the LDH pre-treatment level cut-off value determined with receiver operating characteristics (ROC) curve analysis. Patients were, also, classified according to any variation in LDH serum levels pre- and post-treatment (increased vs. decreased).

The association between categorical variables was estimated by χ^2^ test. Survival distribution was estimated by the Kaplan–Meier method.

Significant differences in probability of survival between the strata were evaluated by log-rank test.

A significant level of 0.05 was chosen to assess the statistical significance.

Cox’s multiple regression analysis was used to assess the role of polymorphisms as prognostic factors adjusted for those variables resulted significant at univariate analysis.

For statistical analysis, overall survival (OS) progression free survival (PFS) were defined as the interval between the date of beginning of sorafenib treatment to death or last follow-up visit, and to clinical progression or death or last follow-up visit if not progressed.

The clinical variables analyzed were: gender (male vs. female), age (≤69 years vs. >69 years), ECOG PS (0 vs. 1–2), Child-Pugh score (A vs. B), BCLC stage (A vs. B-C), median serum α-FP level (≥19 vs. <19), comorbidities (>5%) (cardiovascular, diabetes, other previous neoplasm), etiology (HCV, HBV, Alcoholic, Methabolic-Cryptogenetic), aspartate aminotransferase (AST) serum levels (<UNR vs. ≥UNR upper normal rate 40U/L), alanine aminotransferase (ALT) serum levels (<UNR vs. ≥UNR upper normal rate 40U/L), C reactive protein (CRP) serum levels (<UNR vs. ≥UNR upper normal rate 40U/L), HCV (<5.3 log_10_ IU/mL vs. ≥5.3 log_10_ IU/mL) and HBV (<5.3 log_10_ IU/mL vs. ≥5.3 log_10_ IU/mL) viral loads.

## Results

Seventy-eight patients were available for our analysis: 72 (92%) males and 6 (8%) females. Median age was 69 years (range 51–84) (Table 1). All patients were in Child-Pugh class A. Sorafenib dose reduction was applied in 16 patients (21%) with grade 3 and 4 toxicities. In the general population median PFS was 4.0 months, while median OS was 10.7 months.

**Table 1:** Clinical variables examined

**Clinical variables**		**LDH**	**LDH**	**LDH**	**LDH**	**Total**
		**≤407 U/l**	**>407 U/l**	**Decreased**	**Increased**	
Patients		53 (%)	25 (%)	26	52	78
Gender	Male	52 (98)	21 (84)	24 (92)	49 (94)	73
	Female	1 (2)	4 (16)	2 (8)	3 (6)	5
Median age	< 69	28 (53)	11 (44)	12 (46)	27 (52)	39
	≥ 69	25 (47)	14 (56)	14 (54)	25 (48)	39
ECOG	0	36 (68)	16 (64)	15 (58)	37 (71)	52
	1-2	17 (32)	9 (36)	11 (42)	15 (29)	26
BCLC	B	14 (26)	10 (40)	8 (32)	16 (31)	24
	C	39 (74)	15 (60)	18 (68)	36 (69)	54
Median serum α-FP level	≥ 19 ng/mL	13 (25)	7 (28)	5 (19)	15 (29)	20
	< 19 ng/mL	40 (75)	18 (72)	21 (81)	37 (71)	58
Comorbidities (>5%)	Cardiovascular	11 (21)	4 (16)	5 (19)	10 (19)	15
	Diabetes	6 (11)	2 (8)	3 (12)	5 (10)	8
	Other previous neoplasm	3 (6)	0 (0)	1 (4)	2 (4)	3
Etiology	HCV	20 (38)	9 (36)	10 (38)	19 (37)	29
	HBV	13 (24)	7 (28)	5 (19)	15 (29)	20
	HBV-HCV	2 (4)	0 (0)	1 (4)	1 (2)	2
	Alcoholic	9 (17)	5 (20)	7 (27)	7 (13)	14
	Alcoholic - HBV-HCV	0 (0)	1 (4)	0 (0)	1 (2)	1
	Methabolic-Cryptogenetic	9 (17)	3 (12)	3 (12)	9 (17)	12
Prior locoregional treatments	Yes	25 (47)	13 (52)	15 (58)	23 (44)	38
	No	28 (53)	12 (48)	11 (42)	29 (56)	40
Prior surgery	Yes	11 (21)	4 (16)	6 (23)	9 (17)	15
	No	42 (79)	21 (84)	20 (77)	43 (83)	63
AST	< UNR (40 U/L)	19 (36)	9 (36)	14 (54)	14 (27)	28
	≥ UNR (40 U/L)	34 (64)	16 (64)	12 (46)	38 (73)	50
ALT	< UNR (40 U/L)	23 (43)	11 (44)	9 (35)	25 (48)	34
	≥ UNR (40 U/L)	30 (57)	14 (56)	17 (65)	27 (52)	44
CRP	< UNR (0.5 mg/dL)	11 (21)	4 (16)	6 (23)	9 (17)	15
	≥ UNR (0.5 mg/dL)	42 (79)	21 (84)	20 (77)	43 (83)	63
Viral load HCV RNA	< 5.3 log_10_ IU/mL	7 (35)	5 (56)	3 (30)	9 (47)	12
	≥ 5.3 log_10_ IU/mL	13 (65)	4 (44)	7 (70)	10 (53)	17
Viral load HBC DNA	< 2000 IU/mL	6 (46)	3 (43)	3 (60)	6 (40)	9
	≥ 2000 IU/ml	7 (54)	4 (57)	2 (40)	9 (60)	11

ECOG PS (Eastern Cooperative Oncology Group performance status), BCLC (Barcelona Clinic Liver Cancer), AST (aspartate aminotransferase), ALT (alanine aminotransferase), CRP (C reactive protein), UNR (upper normal rate).

**Figure 1:**
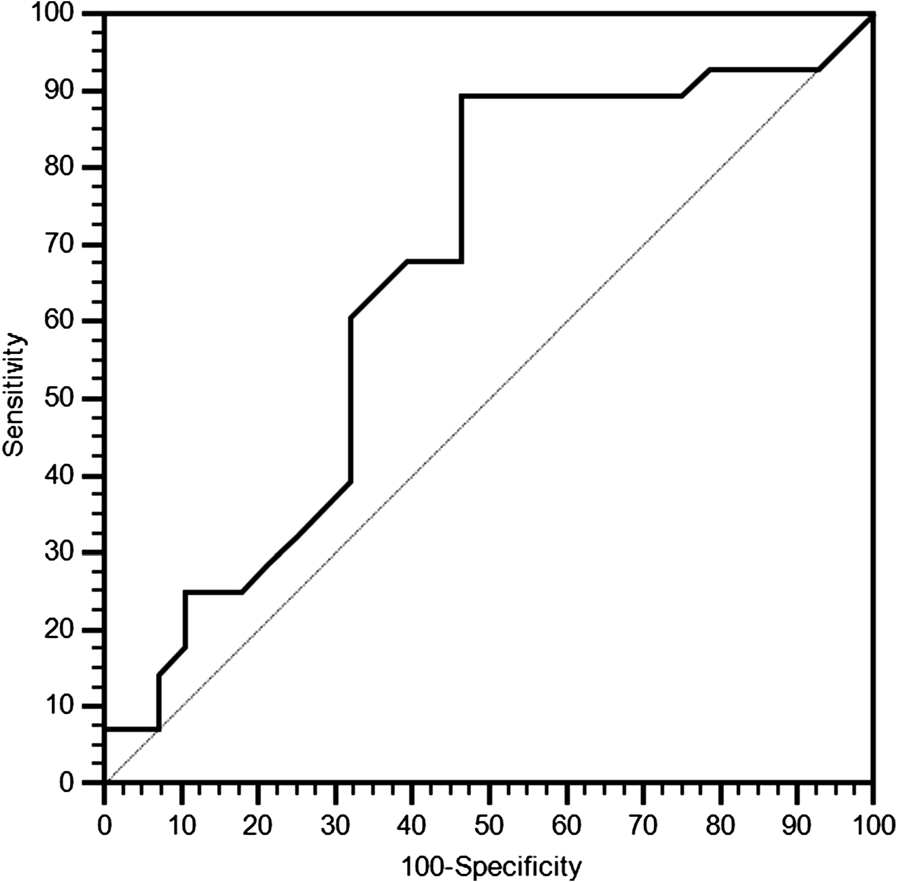
ROC curve for PFS.

**Figure 2:**
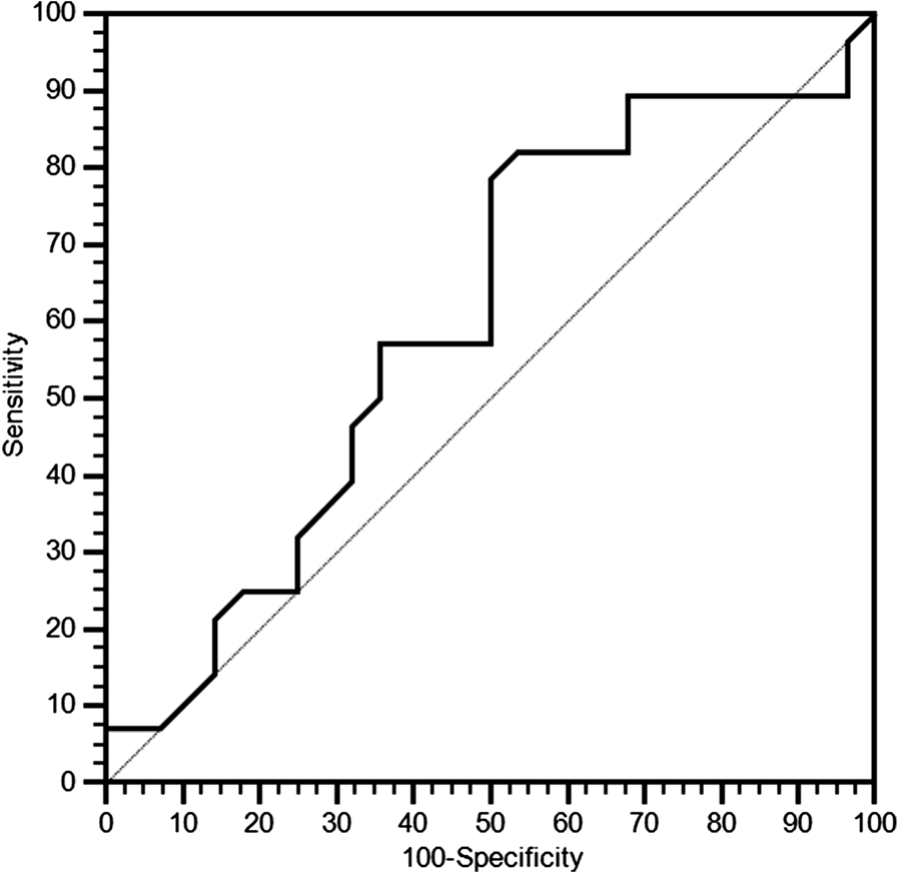
ROC curve for OS.

The cut-off point with the highest sensitivity and specificity for estimating pre-treatment LDH serum levels as a function of treatment clinical activity was set after ROC curve analysis at ≤ 407 U/l both for PFS (Figure 1) and OS (Figure 2).

Fifty-three patients (68%) showed pretreatment LDH serum levels below the cut-off, while 25 (32%) were found above the chosen cut-off. Twenty-six patients (33%) showed decreased LDH serum levels after treatment, while in 52 (67%) this value increased.

**Figure 3:**
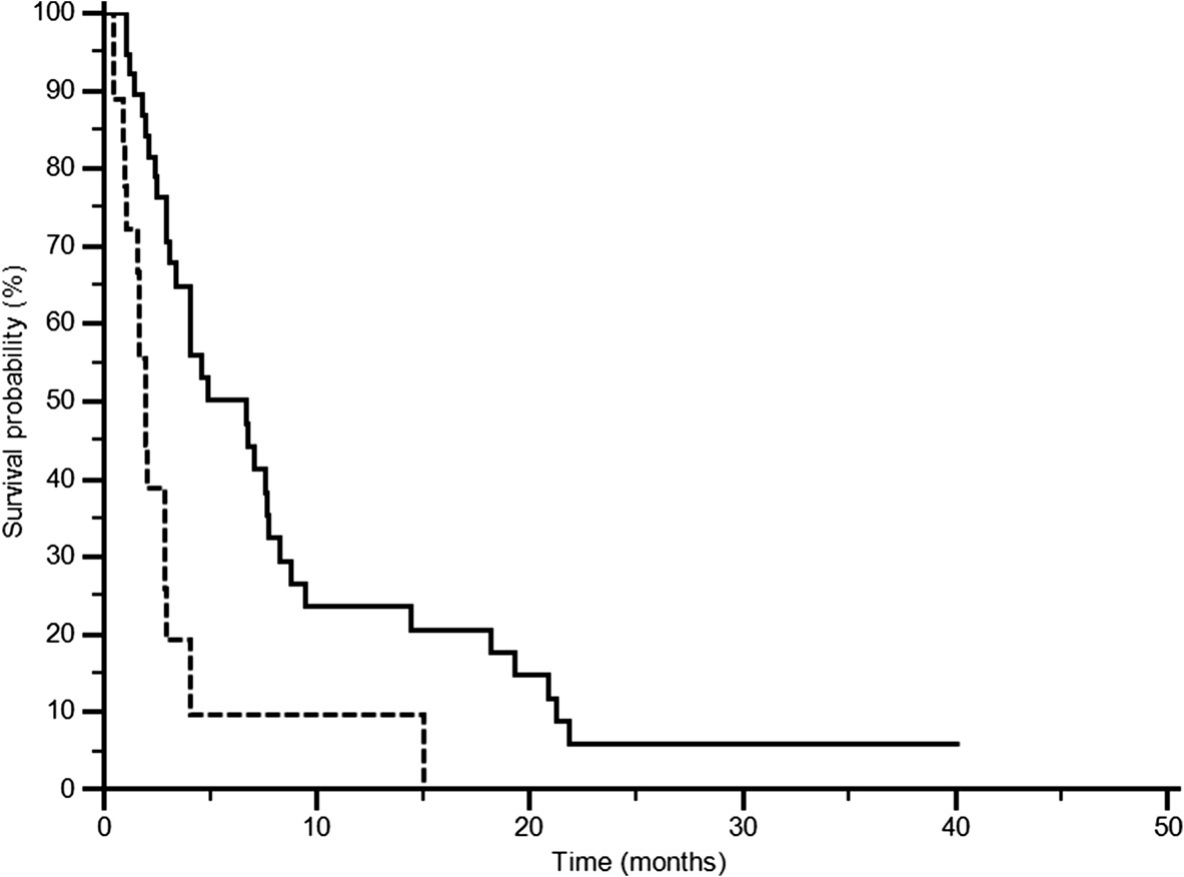
PFS according to LDH pre-treatment: LDH ≤ 407 U/l (———), LDH >407 U/l (--------) (p = 0.0002).

**Figure 4:**
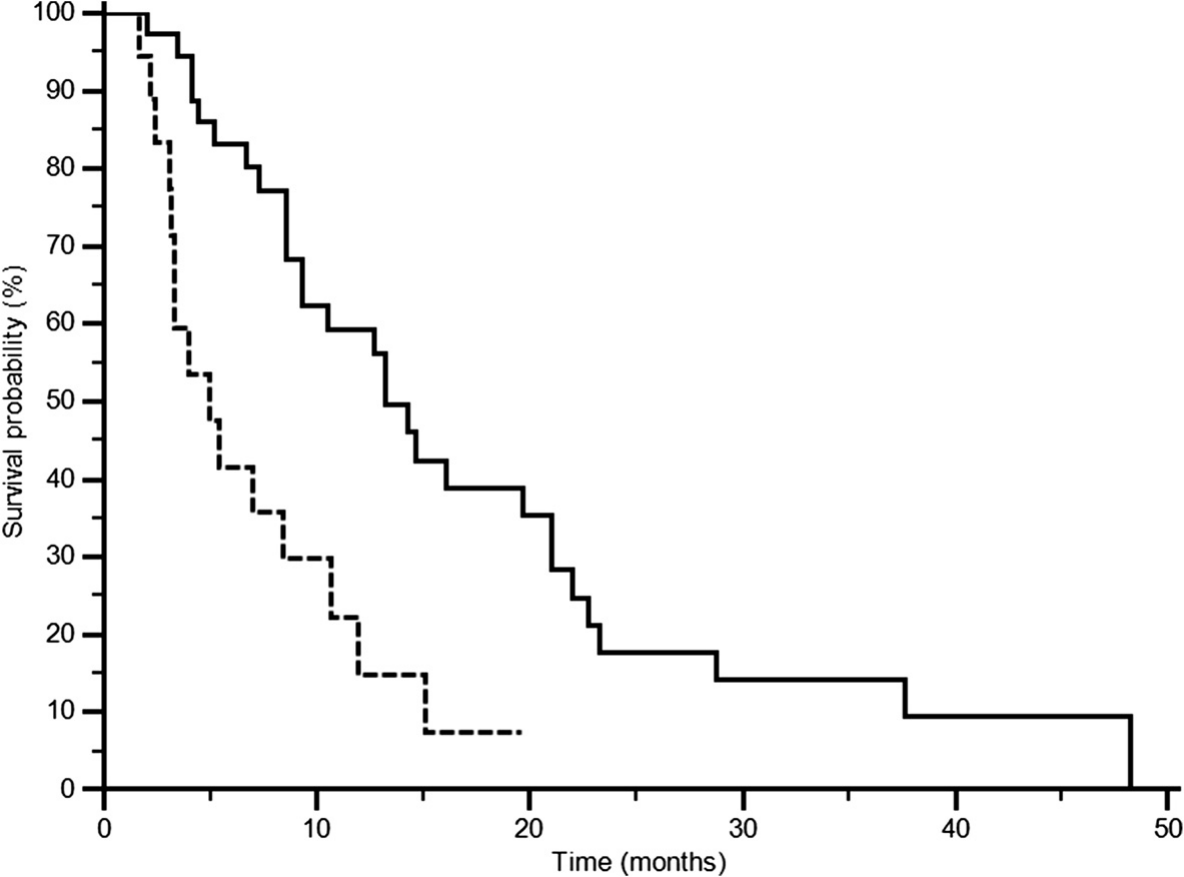
OS according to LDH pre-treatment: LDH ≤ 406 U/l (———), LDH >406U/l (--------) (p = 0.0006).

**Figure 5:**
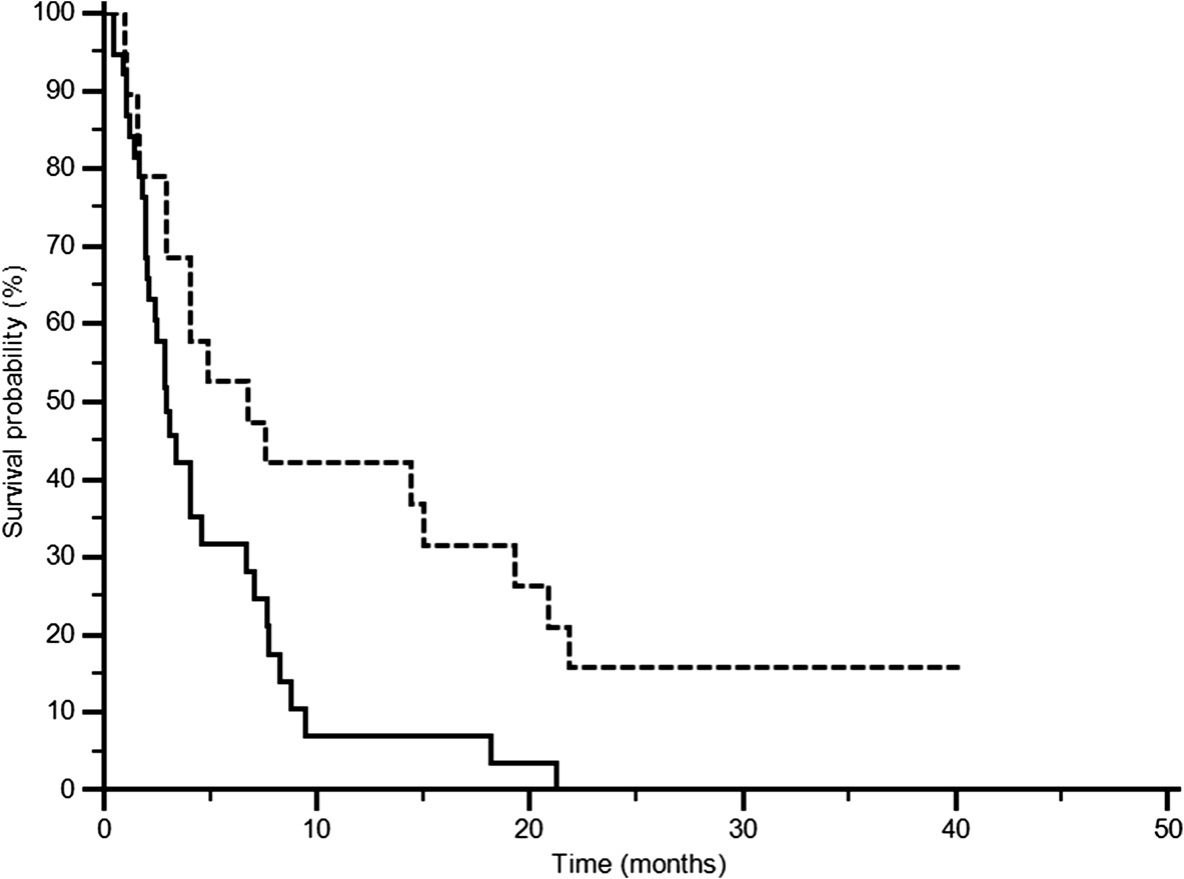
PFS according to LDH variations pre- and post-treatment: increased (———), decreased (--------) (p = 0.0087).

**Figure 6:**
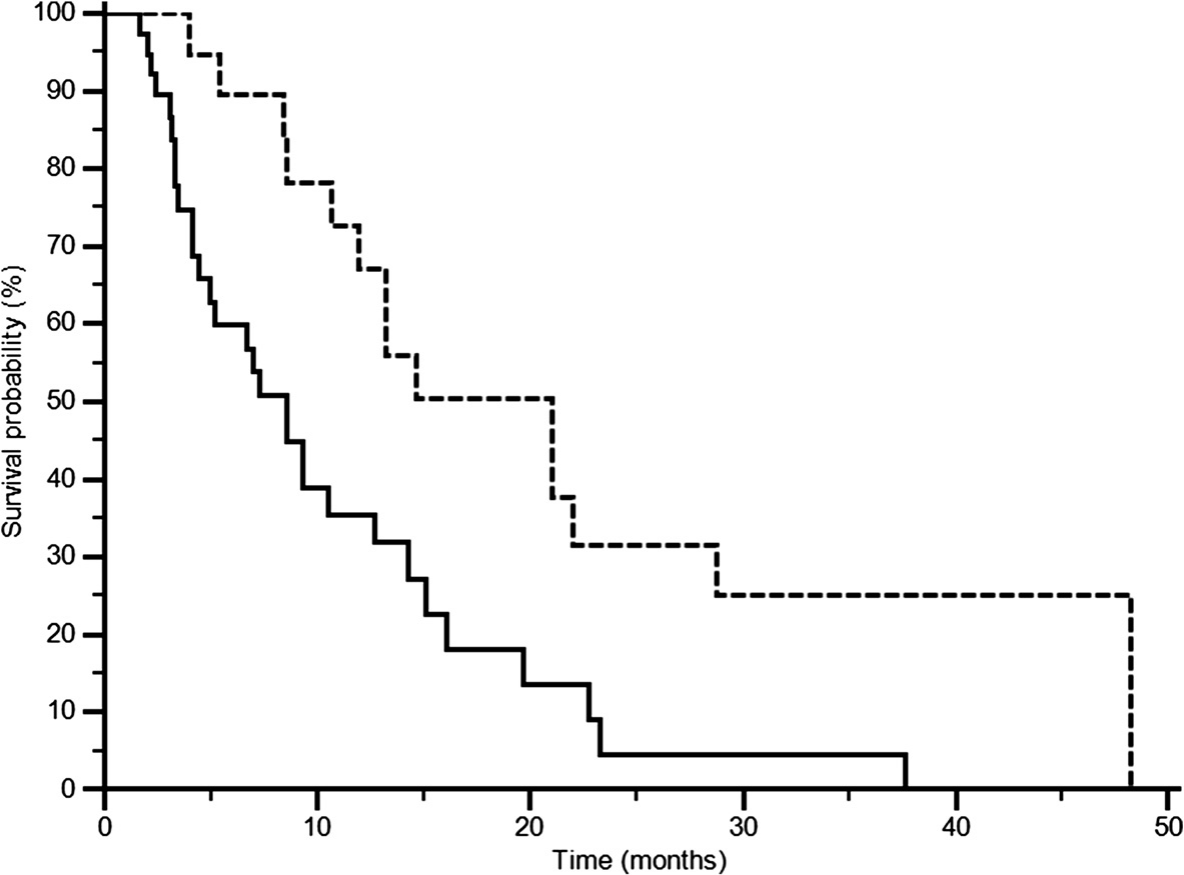
OS according to LDH variations pre- and post-treatment: increased (———), decreased (--------) (p = 0.0035).

At univariate analysis patients with LDH values below the cut-off median PFS was 6.7 months, whereas it was of 1.9 months in patients above the cut-off (p = 0.0002; HR: 2.79; IC: 1.27-6.15) (Figure 3). Accordingly median OS was 13.2 months and 4.9 months in the two groups (p = 0.0006 HR: 2.74; IC: 1.22-6.16) (Figure 4). In patients with decreased LDH values after treatment median PFS was 6.8 months, and median OS was 21.0 months, whereas PFS was 2.9 months and OS was 8.6 in patients with increased LDH levels (PFS: p = 0.0087; HR: 0.48; IC: 0.27-0.84; Figure 5; OS: p = 0.0035; HR: 0.42; IC: 0.23-0.65; Figure 6).

A statistical significant difference in term of PFS and OS was found between patients in B (24 pts) or C (54 pts) stage of BCLC classification (PFS: 7.6 stage B vs. 3.3 stage C, p = 0.0364; OS: 18.4 stage B vs. 9.6 stage C, p = 0.0233).

At multivariate analysis LDH serum levels pre-treatment, the variation post-treatment and BCLC stage emerged as independent prognostic factors predicting outcome in terms of PFS (respectively: p = 0.0197, HR = 0.71; p = 0.0201, HR = 0.19; p = 0.0016, HR = 0.35) and OS (respectively: p = 0.0011, HR = 0.69; p = 0.0039, HR = 0.24; p = 0.0051, HR = 0.39).

No statistically significant differences were found according to other clinical characteristics analyzed (Table 1). Toxicity profiles between patients groups are reported in Table 2.

**Table 2:** Toxicity profile between patients groups

**Clinical variables**		**LDH**	**LDH**	**LDH**	**LDH**	**Total**
		**≤407 U/l**	**>407 U/l**	**Decreased**	**Increased**	
Patients		53 (%)	25 (%)	26	52	78
Any grade toxicity	Global	24 (45)	9 (36)	13 (50)	20 (38)	33
	Rash	5 (9)	3 (12)	4 (15)	4 (8)	8
	Hand-foot	9 (17)	4 (16)	7 (27)	6 (12)	13
	Nausea/vomiting	3 (6)	2 (8)	1 (4)	4 (8)	5
	Diarrhea	11 (21)	3 (12)	6 (23)	8 (15)	14
	Fatigue	5 (9)	5 (20)	4 (15)	6 (12)	10
	Liver dysfunction	0 (0)	2 (8)	1 (4)	1 (2)	2

## Discussion

The introduction of antiangiogenic drugs in HCC treatment saw a wide shift in patients outcome, although a good initial response is observed, frequently this results in a subsequent loss of efficacy. Besides in clinical trials a considerable proportion of patients ranging from 20% to 38% discontinued sorafenib due to adverse events [13-15].

A better selection of patients more likely to benefit from sorafenib treatment, avoiding unnecessary toxicities to those potentially resistant, may be then clinically relevant.

In our experience, LDH serum levels seemed able to predict clinical outcome in terms of PFS and OS for HCC patients treated with sorafenib. We recently reported LDH has a predictive role in terms of PFS and OS in HCC patients treated with transarterial chemoembolization (TACE) [16]. Also Kohles et al. showed a possible prognostic role of pretreatment LDH serum levels in HCC patients undergoing TACE [17], confirming our hypothesis.

These findings are also in accordance with previously published analyses suggesting a relationship between LDH levels and a worse outcome in other tumor types [18].

An increased risk of nodal and distant metastases was correlated with high LDH serum levels and also an high LDH associates with a decreased median overall survival in colorectal cancer patients [19,20].

A strong association has also been demonstrated between the expression of LDH, in particular the LDH-5 isoform and an aggressive phenotype in gastric cancer [21].

Hypoxia and angiogenesis are probably the mechanisms involved in high LDH serum levels and are correlated with enhanced tumour aggressiveness and thus worse prognosis.

Two different clinical trials (CONFIRM 1&2) asserting the efficacy of PTK/ZK (vatalanib), an oral inhibitor of vascular endothelial growth factor (VEGF, in colorectal cancer patients, investigated also the correlation between tumour angiogenesis and LDH levels. Subsequent analyses from these trials in fact evidenced an improved median PFS with the use of PTK/ZK in patients with high serum LDH levels, thus suggesting that tumor angiogenesis represent a key crucial event in presence of high LDH levels [22,23].

In addition to a better prognosis for patients with absolute low LDH level, we demonstrated that a decrease of LDH level during treatment seems to predict a better outcome of HCC patients treated with sorafenb. These findings seem to suggest that the biological phenomenon underlying LDH serum levels is dynamic and may be influenced by medical treatment.

In accordance to this suggestion a single experience of Fiume et al. showed how inhibiting LDH production with oxamic acid in cancer cell lines potentiated the antiproliferative activity of tyrosine kinase inhibitors, such as sorafenib [24].

## Conclusions

We can then speculate that patients with high LDH pretreatment levels may be optimal candidates for clinical trial exploring a multimodality treatment approach.

After confirmation in larger analyses we believe that LDH should be considered as a relevant biological variable to be included in the baseline set up of HCC patients, with the aim to better define the most appropriate therapeutic strategy and to better stratify patients included in clinical trials.

## Abbreviations

LDH: Lactate dehydrogenase; HCC: Hepatocellular carcinoma; TACE: Transarterial chemoembolization; PFS: Progression free survival; OS: Overall survival; TKI: Tyrosine kinase inhibitor; HIF-1: Hypoxia inducible factor 1; AASLD: American Association for the Study of Liver Diseases; α-FP: Alpha-fetoprotein; CT: Computed tomography; MRI: Magnetic resonance imaging; mRESCIST: modified Response Evaluation Criteria in Solid Tumours; ECOG PS: Eastern Cooperative Oncology Group performance status; BCLC: Barcelona Clinic Liver Cancer; IFCC: International Federation of Clinical Chemistry and Laboratory Medicine; ROC: Receiver operating characteristics; VEGF: Vascular endothelial growth factor.

## Competing interests

All authors declare that they have no competing interests.

## Authors’ contributions

FL and BM conception and design, acquisition, analysis and interpretation of data, drafting the manuscript. SM conception and design, acquisition, analysis and interpretation of data, revising the manuscript. SBG, TP acquisition and interpretation of data. GR acquisition and analysis of data, revising the manuscript. DPM, LC and DAM acquisition and analysis of data. DMS and BD acquisition of data. BA revising the manuscript, CS revising the manuscript, final approval of the version to be published. All athors read and approved the final manuscript.

## Pre-publication history

The pre-publication history for this paper can be accessed here:

http://www.biomedcentral.com/1471-2407/14/110/prepub
